# Circulating Betatrophin Is Increased in Patients with Overt and Subclinical Hypothyroidism

**DOI:** 10.1155/2016/5090852

**Published:** 2016-04-26

**Authors:** Cheng Han, Xinghai Xia, Aihua Liu, Xiaowen Zhang, Mi Zhou, Chuhui Xiong, Xin Liu, Jie Sun, Xiaoguang Shi, Zhongyan Shan, Weiping Teng

**Affiliations:** ^1^Department of Endocrinology and Metabolism, Institute of Endocrinology, Liaoning Provincial Key Laboratory of Endocrine Diseases, The First Affiliated Hospital of China Medical University, Shenyang, Liaoning 110001, China; ^2^Department of Cellular and Molecular Physiology, College of Medicine, Pennsylvania State University, Hershey, PA 17033, USA

## Abstract

Thyroid hormone (TH) affects many metabolic processes such as promoting oxidation of sugar, fat, and protein in many tissues. Thyroid dysfunction is associated with metabolic disorders. The newly discovered adipocyte- and hepatocyte-derived cytokine, betatrophin, has been reported to be involved in metabolic diseases, but its influence on thyroid dysfunction is uncertain. Therefore, the present study aims to evaluate circulating betatrophin levels in subjects with different thyroid function status and to predict the factors associated with betatrophin levels, especially whether thyroid stimulating hormone (TSH), TH, or thyroid autoantibodies are associated with betatrophin levels. In the study, serum betatrophin was measured in the subjects grouped as overt hypothyroidism (OH), subclinical hypothyroidism (SCH), euthyroid with isolated thyroid peroxidase antibody positivity (isolated Ab), and healthy control (HC), according to their thyroid functions. From our results, we found that betatrophin may be associated with thyroid insufficiency but not thyroid autoimmunity. Thus, when interpreting the results of betatrophin, thyroid functions should also be taken into consideration.

## 1. Introduction

Currently, multiple independent studies have characterized a novel protein predominantly expressed in liver and white adipose tissue named betatrophin (alternatively called lipasin, refeeding induced in fat and liver or angiopoietin-like protein 8) [1, 2]. Recent studies suggest that betatrophin may help balance energy metabolism and pancreatic cell proliferation presumably through its functions as an endocrine hormone in human and murine models [3]. Indeed, adenovirus-mediated hepatic overexpression of betatrophin increases serum triglyceride (TG) in mice [4], whereas betatrophin knockout mice had disrupted TG metabolism which was manifested as decreased adipose tissue mass and a pronounced reduction in serum TG in the fed state [5]. Also, when betatrophin was overexpressed in a mouse model, it dramatically and specifically induced pancreatic *β*-cell proliferation and improved glucose tolerance. Thus, betatrophin was concluded to play a vital role in maintaining normal glucose tolerance and insulin sensitivity [1]. These reports suggest that betatrophin function is mediated by complex regulatory processes. However, the precise physiological role is still unclear. As betatrophin has a dual role in both lipid metabolism and glucose homeostasis, it may be a promising metabolic regulator worthy of further research [2, 3].

Hypothyroidism is the result of inadequate production of thyroid hormone or inadequate action of thyroid hormone in target tissues. Primary hypothyroidism is the principal manifestation of hypothyroidism, but other causes include central deficiency of thyrotropin-releasing hormone or thyroid-stimulating hormone (TSH), or consumptive hypothyroidism from excessive inactivation of thyroid hormone, which may lead to severe human health crisis [6–10]. About 50% of clinical hypothyroidism cases are of autoimmune origin [11]. Thyroid hormone affects many metabolic processes by promoting oxidation of sugar, fat, and protein in various tissues [12, 13]. Thus, it is a regulator of metabolism and energy homeostasis that modifies growth, body weight, and thermogenesis [13]. Hypothyroidism is also documented to be related to metabolism disorders [12, 14, 15]. The levels of circulating adipocyte- and hepatocyte-derived metabolic regulators, such as leptin, fibroblast growth factor 21 (FGF21), adiponectin, retinol-binding protein-4, and resistin, were reported to alter in patients with thyroid dysfunctions [16–18].

Despite these findings and the well-established link between metabolic disorders and thyroid dysfunctions, few clinical studies have reported the potential association of betatrophin with thyroid disease. In general, autoimmune thyroiditis progresses through three clinical stages that are defined by thyroid function: the early stage (euthyroid with positive autoantibodies), the second stage (subclinical hypothyroidism), and the final stage (overt hypothyroidism). It has previously been found that these clinically defined disease stages are closely correlated to the severity of the disease. Therefore, the present study aims to evaluate circulating betatrophin levels in subjects with different thyroid function status and to predict the factors associated with betatrophin levels, especially whether thyrotropin, thyroid hormone, or thyroid autoantibodies are associated with betatrophin levels.

## 2. Materials and Methods

### 2.1. Subjects

This study was conducted at the Department of Endocrinology and Metabolic Disease at the First Hospital Affiliated to China Medical University from June 2012 to November 2014. All research protocols were approved by the Medical Ethics Committee of China Medical University and were congruent with the Declaration of Helsinki. All participants were provided with written informed consent after the research protocols were carefully explained to them. A total of 122 subjects were included from healthy volunteers and patients attending the Department of Endocrinology and Metabolic Disease at the First Hospital Affiliated to China Medical University. Age-, sex-, and body mass index- (BMI-) matched subjects with overt hypothyroidism (OH, *n* = 31), subclinical hypothyroidism (SCH, *n* = 30), isolated thyroid peroxidase antibody (TPOAb) positivity (isolated Ab, *n* = 30), and healthy controls (HC, *n* = 31) were included after excluding identified acute or chronic illness, such as diabetes mellitus, acute-chronic renal disease, nephritic-range proteinuria, coronary heart disease, heart failure, peripheral artery disease, cerebrovascular event, malignancy, liver diseases, rheumatic diseases, alcohol intake, and smoking. The subjects with overt hypothyroidism, subclinical hypothyroidism, and isolated thyroid peroxidase antibody positivity were newly diagnosed but not started on a treatment yet.

### 2.2. Diagnostic Criteria

Thyroid function reference ranges were provided by measurement kit manufacturers: TSH 0.35–4.94 mIU/L, FT4 9.01–19.05 pmol/L, TPOAb 0–5.6 IU/mL, and thyroglobulin antibody (TgAb) 0–4.11 IU/mL. For our study, OH was defined as TSH > 4.94 mIU/L and FT4 < 9.01 pmol/L, SCH was defined as TSH > 4.94 mIU/L and a FT4 within the normal range, isolated Ab was defined as an isolated TPOAb > 5.61 IU/mL with a normal TSH and FT4, and HC was defined as TSH, FT4, TPOAb, and TgAb in normal ranges by the assay kits.

### 2.3. Anthropometric and Biochemical Measurements

Heights and weights were measured to the nearest 0.1 cm and 0.1 kg, respectively, and BMI was calculated as weight divided by height squared (kg/m^2^). At that time, a venous blood sample was taken after overnight fasting. Blood was allowed to coagulate at 4°C and specimens were separated by centrifugation for 15 min at 3,000 rpm. Serum samples were subsequently stored in aliquots without preservative at −80°C for about 3 months until betatrophin measurement. Serum TSH, FT4, free tri-iodothyronine (FT3), TPOAb, and TgAb were tested with a chemiluminescence immunoassay (ARCHITECT system i2000SR, Abbott Laboratories). Then, alanine aminotransferase (ALT), aspartate aminotransferase (AST), gamma glutamyl transpeptidase (GGT), low density lipoprotein cholesterol (LDL-C), high density lipoprotein cholesterol (HDL-C), total cholesterol (TC), and TG were measured using a Cobas Elecsys 601 (Roche Diagnostics, Switzerland).

### 2.4. Measurement of Betatrophin

Fasting serum betatrophin was measured with ELISA kits (EIAab Science, Wuhan, China, Catalog number E11644h) with an intra-assay coefficient of variation (CV) of ≤4.8% and an interassay CV of ≤7.2%. The procedures were performed in accordance with the manufacturer's instructions. All samples were analyzed in duplicate and if duplicates were >15% CV, the sample measurement was repeated.

### 2.5. Statistical Analysis

Data were expressed as means ± standard deviations (S.D.) for normally distributed variables, medians with interquartile range for nonnormally distributed variables, and frequencies for categorical variables. For comparisons among study groups, normally distributed variables were assessed using one-way ANOVA followed by Bonferroni correction in paired comparisons. Variables failed the normality test, so these variables were assessed using Kruskal-Wallis one-way ANOVA for ranks in groups and pairwise comparisons were performed using the Mann-Whitney rank sum test. Pearson's Chi-square test was used to compare sex ratios and correlation analysis was performed using the Pearson correlation or Spearman rank correlation method. To identify independent relationships and adjust for effects of covariates, multiple linear regression analyses were performed. Nonnormally distributed variables were analyzed after logarithmic transformation (Log). All calculated *P* values were two-sided, and *P* values < 0.05 were considered statistically significant. All statistical analyses were performed with SPSS software (version 20.0).

## 3. Results

### 3.1. Circulating Betatrophin Was Elevated in Patients with Hypothyroidism

**Figure 1:**
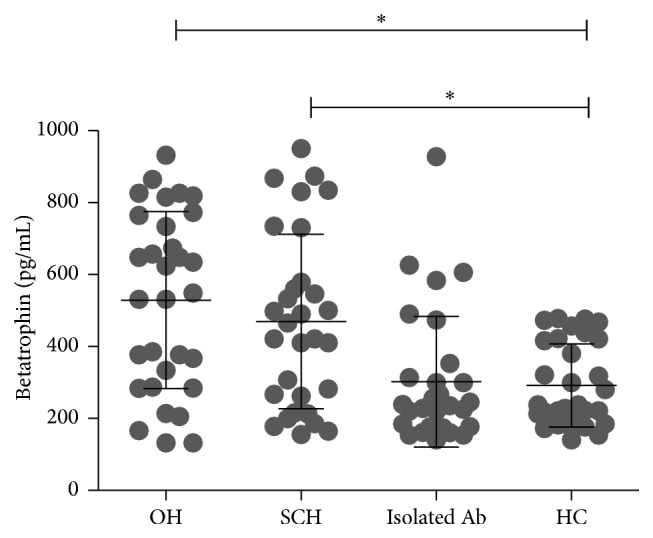
Serum betatrophin was significantly elevated in the OH group compared with the HC group. Also, betatrophin was higher in the SCH group than in the HC group. Besides, betatrophin was not different between the isolated Ab group and the HC group.  ^*∗*^Statistically significant difference of betatrophin levels between the two groups.

Characteristics of the subjects in different groups are described in Table 1. Significant differences existed in TSH, FT4, FT3, TPOAb, TgAb, LDL-C, and TG among the groups (all *P* values < 0.05). There were no significant differences in age, sex, BMI, diastolic blood pressure (DBP), systolic blood pressure (SBP), HDL-C, TC, and liver function among the groups (all *P* values > 0.05).  Figure 1 depicts serum betatrophin being significantly elevated in the OH group compared with the HC group (548.9 [287.0–764.2] versus 238.0 [184.3–421.4] pg/mL; *P* = 0.004). Also, betatrophin was higher in the SCH group than in the HC group (443.3 [250.5–616.6] versus 238.0 [184.3–421.4] pg/mL; *P* = 0.033). Betatrophin was higher in OH group than in the isolated Ab group (548.9 [287.0–764.2] versus 237.3 [176.7–323.8]; *P* = 0.001), and betatrophin was elevated in SCH group compared with the isolated Ab group (443.3 [250.5–616.6] versus 237.3 [176.7–323.8]; *P* = 0.027). Besides, betatrophin was not different between the isolated Ab group and the HC group (237.3 [176.7–323.8] versus 238.0 [184.3–421.4] pg/mL; *P* > 0.05).

### 3.2. Betatrophin Correlates with Thyroid Function

**Figure 2:**
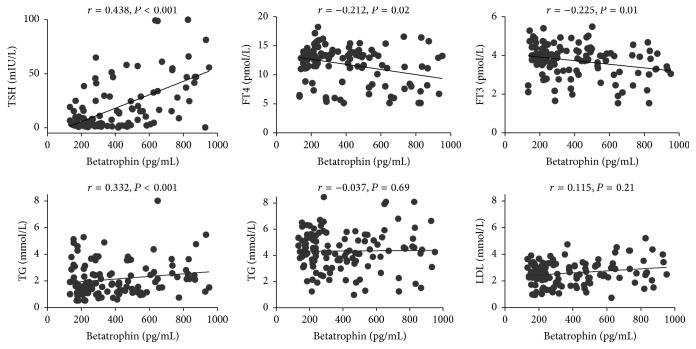
Betatrophin was negatively correlated with FT4 and FT3 but positively correlated with TSH and TG.

Figure 2 depicts a correlation analysis between the biochemical variables and betatrophin. Betatrophin was negatively correlated with FT4 (*r* = −0.212, *P* = 0.02) and FT3 (*r* = −0.225, *P* = 0.01) but positively correlated with TSH (*r* = 0.438, *P* < 0.001) and TG (*r* = 0.332, *P* < 0.001). It was also shown that betatrophin had no correlation with TC and LDL-C. To further control the confounding factors, a multivariate regression analysis was applied.  Table 2 depicts TSH (*β* = 0.160, *P* < 0.001) and TG (*β* = 0.198, *P* = 0.026) as independent predictors of betatrophin after adjustments for FT4, FT3, GGT, BMI, and Age.

## 4. Discussion

To our knowledge, this is the first study to evaluate an association between betatrophin and thyroid dysfunction. Here, we report that circulating betatrophin was significantly increased in the OH and SCH groups compared to the isolated Ab and HC groups. In addition, we observed that serum betatrophin level was increased in correlation with increased severity of hypothyroidism (with increased TSH and decreased FT4). Also, serum betatrophin concentrations were independently associated with TSH and TG.

Since the incidences of metabolic diseases are increasing rapidly, more and more research interests are focused on them [19, 20]. Recently, several independent groups have identified and characterized a liver- and fat-derived hormone named betatrophin within human and murine models. Betatrophin is credited for being a metabolic regulator, capable of influencing lipid and glucose metabolism [1, 5]. Different studies have shown that betatrophin was likely associated with multiple metabolic disorders. Previous studies in vitro and in mouse models have demonstrated that mouse body weight and fat mass as well as serum TG and NEFA (nonesterified fatty acid) levels were reduced in betatrophin-null mice, whereas serum cholesterol, plasma glucose (fasted and refed), and insulin levels were not significantly altered, compared with wild-type littermates [5, 21]. Quagliarini's group further clarified that the betatrophin-induced serum TG content was angiopoietin-like protein 3 (ANGPTL3) dependent and coexpression of betatrophin with ANGPTL3 further increased the serum TG level [22]. In vivo, accumulating evidence was provided by scholars to describe the association between betatrophin and lipid profile. Fenzl's group suggested that betatrophin was positively correlated with atherogenic lipids in 19 morbidly obese individuals and 18 type 2 diabetic individuals [23]. Further studies indicated that, in humans, betatrophin sequence variations were associated with differences in lipids [24, 25]. In addition, Yi and coworkers confirmed that transient expression of betatrophin in mouse liver significantly promoted pancreatic *β*-cell proliferation, expanded *β*-cell mass, and improved glucose tolerance [1]. To further study the roles of betatrophin in diabetes and other metabolic diseases, many clinical researches have been done and the results are controversial. These studies show that betatrophin levels were altered in various physiologic conditions, such as the postprandial state [26], and pathological conditions, such as type 2 diabetes [26–30], type 1 diabetes [30, 31], and obesity [26, 29], and were associated with metabolic parameters, such as BMI [26, 29], glucose [26, 28], and insulin resistance [28, 29]. Recently, based on the findings of a meta-analysis, circulating betatrophin level of T2DM patients is higher than that of nondiabetic adults in the nonobese population, but not in the obese population [32]. Hence, it is believed that betatrophin may have a dual role in mediating both triacylglycerol metabolism and glucose homeostasis and may have effects on multiple metabolic diseases.

It has been well established that overt hypothyroidism is associated with metabolic syndrome such as atherosclerotic cardiovascular disease, obesity, insulin resistance/diabetes, and dyslipidemia [15]. However, subclinical hypothyroidism is relatively more common, occurring in 4–10% of the adult population [33]. In addition, it is estimated that 1 to 11% of all patients with dyslipidemia have subclinical hypothyroidism [34]. Many metabolic regulators and adipokines/hepatokines including leptin, adiponectin, resistin, and FGF21 have been found to be altered with thyroid dysfunction [16]. These metabolic regulators may have similar functions with betatrophin. Still, because the association between betatrophin and hypothyroidism is not clear, we sought to better understand the potential role of betatrophin in hypothyroidism. However, up to date, the reason for elevated betatrophin in subjects with overt hypothyroidism and subclinical hypothyroidism is still unclear. Consistent with our study, many epidemiological studies demonstrated that subclinical hypothyroidism may worsen the serum lipid profiles. TG levels were found with higher levels in subclinical hypothyroid subjects than in euthyroid subjects in a study among 25,862 participants in Colorado [35]. Similar results were also found in a study among 1534 Chinese adults [36]. Currently, the effects of L-T4 therapy on serum lipids in subclinical hypothyroidism have been controversial. There is general consensus that individuals with serum TSH values greater than 10 mIU/liter should receive L-T4 treatment, but published opinions regarding less severe subclinical hypothyroidism vary [37, 38]. In our study, we have found that there is an association between subclinical hypothyroidism and betatrophin. Increased betatrophin levels and the positive relationship between circulating betatrophin and triglycerides levels could be detected also in subclinical hypothyroid patients, suggesting that hypertriglyceridemia in mild hypothyroidism might be mediated by increased betatrophin release. As a result, when considering the relationship between betatrophin and increased TG, all the evidences together make us think of whether betatrophin may act as a promising therapeutic target of SCH patients accompanied with hyperlipidemia. It still acts as a direction worth more effort.

There are some limitations of our study. First, it was a cross-sectional study so only associations (not causation) in serum betatrophin and thyroid function could be addressed. Moreover, we allotted subjects into groups according to thyroid function prior to random selection which may have introduced selection bias. Based on the limitations above, more large-scale population-based prospective studies are needed.

In conclusion, we report that circulating betatrophin was elevated in patients with OH and SCH. Serum TSH was independently associated with betatrophin. Thus, it seems that thyroid insufficiency but not thyroid autoimmunity may have effect on the level of serum betatrophin. As betatrophin has attracted more and more attention in the studies of metabolic diseases, we suggest that thyroid hormones should be considered when evaluating betatrophin. Further large-scale prospective studies are needed to elucidate the role of betatrophin in the development of hypothyroidism.

## Figures and Tables

**Table 1:** Characteristics of the study population.

	OH	SCH	Isolated Ab	HC	*P *value
Sex (F/M)	31 (25/6)	30 (26/4)	30 (25/5)	31 (25/6)	0.910
Age (years)^a^	49.0 ± 14.1	51.8 ± 18.2	48.0 ± 13.2	52.0 ± 11.1	0.617
BMI (kg/m^2^)^a^	20.3 ± 1.3	20.1 ± 1.5	20.3 ± 1.6	20.0 ± 1.4	0.850
SBP (mmHg)^a^	121.6 ± 6.1	124.0 ± 6.1	124.2 ± 5.7	121.5 ± 7.7	0.192
DBP (mmHg)^a^	72.5 ± 8.3	71.8 ± 6.7	72.0 ± 4.3	70.0 ± 5.0	0.429
TSH (mIU/L)^b^	34.0 (19.3–57.2)	15.5 (10.9–40.4)	3.0 (2.2–3.8)	1.8 (1.3–3.4)	<0.001
FT4 (pmol/L)^b^	6.7 (5.7–8.2)	13.3 (11.5–13.6)	13.4 (12.4–14.8)	13.5 (12.9–14.7)	<0.001
FT3 (pmol/L)^b^	3.0 (2.2–3.3)	4.0 (3.6–4.1)	4.0 (3.8–4.3)	4.3 (3.8–4.7)	<0.001
TPOAb (IU/mL)^b^	1000.0 (500.0–1000.0)	771.3 (581.7–1000.0)	1000.0 (781.2–1000.0)	0.4 (0.2–1.2)	<0.001
TgAb (IU/mL)^b^	379.9 (75.2–1000.0)	169.2 (46.6–1000.0)	110.8 (55.3–521.5)	1.1 (0.9–1.6)	<0.001
GGT (IU/L)^b^	18 (12–31)	17 (13–25)	16 (10–26)	15 (10–25)	0.395
AST (IU/L)^b^	18 (16–22)	18 (15–21)	17 (14–19)	18 (15–23)	0.631
ALT (IU/L)^b^	15 (11–20)	14 (11–40)	15 (10–17)	16 (10–21)	0.657
HDL-C (mmol/L)^b^	1.4 (1.2–1.7)	1.2 (1.0–1.6)	1.3 (1.1–1.5)	1.4 (1.2–1.9)	0.154
LDL-C (mmol/L)^b^	3.0 (2.5–3.4)	2.5 (2.2–3.3)	2.3 (1.7–3.3)	2.4 (1.6–2.8)	0.005
TG (mmol/L)^b^	2.2 (1.2–2.6)	1.7 (1.4–2.4)	1.3 (1.1–2.6)	1.3 (0.9–1.8)	0.024
TC (mmol/L)^b^	4.6 (3.7–5.6)	4.2 (2.9–5.8)	4.3 (3.9–5.3)	3.8 (3.4–4.8)	0.083
Betatrophin (pg/m)^b^	548.9 (287.0–764.2)	443.3 (250.5–616.6)	237.3 (176.7–323.8)	238.0 (184.3–421.4)	<0.001

F: female; M: male.

^a^Results are shown as arithmetic mean ± S.D.

^b^Results are shown as median with interquartile range.

**Table 2:** Variables independently associated with betatrophin, as identified by linear regression analysis^a,b^.

Variables	*β*	SE	*P*
Log TSH	0.160	0.045	<0.001
Log TG	0.198	0.088	0.026

^a^Variables included in the original model were according to both statistical significance and clinical significance, including TSH, FT4, FT3, TG, GGT BMI, and Age.

^b^Nonnormally distributed variables such as betatrophin, TSH, FT4, FT3, TG, and GGT were logarithmically transformed before testing.
